# Computational modeling of locoregional recurrence with spatial structure identifies tissue-specific carcinogenic profiles

**DOI:** 10.3389/fonc.2023.1116210

**Published:** 2023-04-06

**Authors:** Sharafudeen Dahiru Abubakar, Mitsuaki Takaki, Hiroshi Haeno

**Affiliations:** ^1^ Research Institute for Biomedical Science, Tokyo University of Science, Noda, Japan; ^2^ Department of Computational Biology and Medical Sciences, Graduate School of Frontier Sciences, The University of Tokyo, Chiba, Japan

**Keywords:** computational modeling, cancer initiation, cancer recurrence, field cancerization, stochastic processes

## Abstract

**Introduction:**

Local and regional recurrence after surgical intervention is a significant problem in cancer management. The multistage theory of carcinogenesis precisely places the presence of histologically normal but mutated premalignant lesions surrounding the tumor - field cancerization, as a significant cause of cancer recurrence. The relationship between tissue dynamics, cancer initiation and cancer recurrence in multistage carcinogenesis is not well known.

**Methods:**

This study constructs a computational model for cancer initiation and recurrence by combining the Moran and branching processes in which cells requires 3 or more mutations to become malignant. In addition, a spatial structure-setting is included in the model to account for positional relativity in cell turnover towards malignant transformation. The model consists of a population of normal cells with no mutation; several populations of premalignant cells with varying number of mutations and a population of malignant cells. The model computes a stage of cancer detection and surgery to eliminate malignant cells but spares premalignant cells and then estimates the time for malignant cells to re-emerge.

**Results:**

We report the cellular conditions that give rise to different patterns of cancer initiation and the conditions favoring a shorter cancer recurrence by analyzing premalignant cell types at the time of surgery. In addition, the model is fitted to disease-free clinical data of 8,957 patients in 27 different cancer types; From this fitting, we estimate the turnover rate per month, relative fitness of premalignant cells, growth rate and death rate of cancer cells in each cancer type.

**Discussion:**

Our study provides insights into how to identify patients who are likely to have a shorter recurrence and where to target the therapeutic intervention.

## Introduction

Cancers are dynamic cells whose features favors cellular proliferation, differentiation and movement while restricting cell death and tissue stability (1). Surgery is a potent curative tool for managing cancers, however, local recurrence has remained a clinically significant problem in most cancer types (2–4). Local recurrence rates could be as high as about 85% (5) for ovarian cancer, or 30% in non-small cell lung cancer (NSCLC) (6, 7) and as low as between 8% (8) to 16.5% (9) in breast cancer. Advanced surgical techniques, chemotherapy, radiotherapy (4) as well as endocrine therapy (10) are being used to minimize locoregional recurrence but “minimal” improvements and treatment-related mortality has highlighted the need for a better understanding and strategy for local recurrence (11, 12).

Since its introduction in 1953 (13), field cancerization has been recognized as a major cause of local recurrence (14). Field cancerization is the presence of “histologically normal” cells surrounding cancer cells that have acquired some but not all the genetic and phenotypic traits required for malignancy in a tissue (15, 16). These cancerized cells may have a survival or growth advantage and does serve as a hotbed for recurrent tumors as only a small number of additional steps are needed for cancer initiation. Recent advances in molecular, genomic and bulk sequencing techniques have supported the role of field cancerization (17). In breast cancer, microsatellite markers, epigenetic aberrations, transcriptomic deregulations and hTERT overexpression have been detected in histologically normal mammary tissues (18, 19). In head and neck cancer, loss of heterozygosity of chromosome 9p and telomere dysregulation were commonly observed in benign squamous hyperplasia (20, 21). In colon cancer patients with Crohn’s ileocolitis, the same mutations of KRAS, CDKN2A, and TP53 were observed within neoplasia and non-tumor epithelium (22, 23). In Non-small cell lung cancer, miRNA dysfunction has been shown at the level of the tumor and cancerized field (24). Residual tumor (25), anesthesia choice (26) and CTCs (27) have been shown to have minimal impact on cancer recurrence; therefore, a proper understanding of the field cancerization formation process will contribute to the estimation of the risk of locoregional recurrence and the development of optimal treatment in each tissue.

Several Theoretical studies have shed light on field cancerization impacts on cancer initiation (15). Jeon et al. examined the multistage clonal expansion model by employing the Poisson process to consider the effects of premalignant cells on cancer initiation (28). The model was applied clinically to predict the long-term impact of ablative treatments on reducing esophageal adenocarcinoma incidence in Barrett’s esophagus (29). Foo et al. developed a spatial evolutionary framework to determine the size distribution of histologically undetectable premalignant fields during diagnosis (30). This model was applied to the head and neck cancer and revealed that the patient’s age was a critical predictor of the size and multiplicity of precancerous lesions (31). These findings are in agreement with bulk sequencing data that shows the accumulation of cancer-related mutations as we age (32). A 2-step tumor initiation model provides insights into the relationship between different tissue kinetic parameters and the incidence of recurrent cancers (33) by using public datasets from the cancer genome atlas (TCGA), a valuable resource for genomic and clinical data analysis (34, 35) but fails to account the varying number of mutational hits required for carcinogenesis (36–38). The cancer genome atlas (TCGA) is a rich computational resource for the genomic and mutational data for different cancer types (34, 35) and will be helpful in validating our understanding of field cancerization.

This study developed a novel computational model of multi-stage cancer initiation and recurrence with spatial structure. We employed a combined stochastic model of Moran and a branching process to represent tissue and tumor dynamics, respectively, in order to observe cancer initiation and relapse after surgical resection of the first tumor *in silico*. Particularly, we focused on the relationship between the tissue compositions at the time of surgery and the time until the emergence of recurrent tumors. This model builds upon our previous work (33) by expanding the number of mutation steps for carcinogenesis *via* adding cell types as well as incorporating the spatial structure setting. Moreover, based on the public clinical datasets for locoregional recurrence rates, we succeeded in identifying tissue-specific carcinogenic parameters for various cancer types. Our approach provided insights on how to predict the time of recurrence from the tissue dynamics at the time of surgery and how to intervene patients to prevent the recurrence.

## Materials and methods

### Computational model

**Figure 1:**
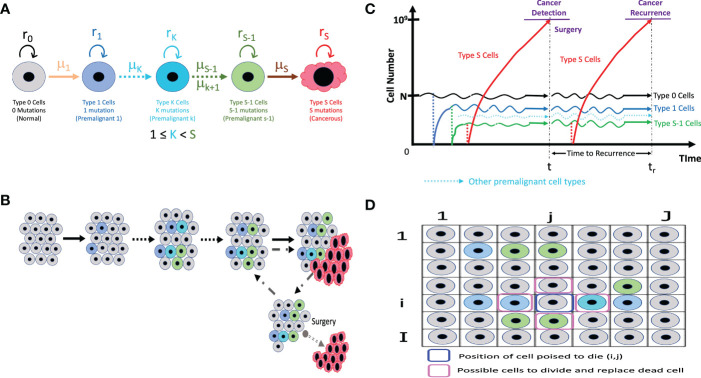
The illustrative representation of our models **(A)** The different cell types in our models with its own mutation rate (*µ*) and fitness (*r*). Type *K* cells may not be applicable if only 3 mutations or less are needed for carcinogenesis. **(B)** In a normal tissue composed of Type 0, Type 1, Type *K*, and Type *S*-1 cells, cell turnover is conducted according to the Moran process, and the number of cells is kept constant. If a Type *S* cell emerges, it proliferates without limit and can be detected and primed for surgery when the cancer cell number reaches 10^9^. **(C)** At surgical intervention, all the Type *S* cells are resected while the number of Type 0, Type 1, Type *K* (if present) and Type *S*-1 cells remaining in a tissue are preserved. The time until the next Type *S* population reaches 10^9^ is measured as time to recurrence. **(D)** The spatial structure integration in the model accounts for the positional relation between a cell poised to die and the possible cells that can divide to replace the dead cells.

This model employs the multi-stage carcinogenesis concept. As tissues might require anywhere between 2 to 8 driver mutations (denoted by S) for malignant transformation (37), we first identify different cell types that can lead to a malignant transformation based on number of driver mutations. Let us visualize the dynamics of 5 types of cells in a tissue (**Figure 1**). “Type 0”, “Type 1”, “Type *K*”, “Type *S*-1” and “Type *S*” represent normal healthy cells with no mutation; premalignant cells with one cancer-related mutation; premalignant cells with *K* cancer-related mutation, premalignant cells with *S*-1 cancer-related mutations and cancer cells with *S* cancer-related mutations, respectively. Emergence of cancer cells must be preceded by that of premalignant cells with mutations from Type 1 cell to Type *S*-1 cell. Type *K* cell may or may not be present depending on number of mutations required for carcinogenesis. We assume that a normal healthy tissue consists of Type 0, Type 1, Type *K* and Type *S*-1 cells undergoing cellular turnover with a small probability of a mutation. Moran process is employed to consider the tissue turnover dynamics, where the total number of Type 0, Type 1, Type *K* and Type *S*-1 cells is kept constant as *N* (39). The turnover rate of a whole tissue is defined by *d*. Type *S* cells are considered as uncontrolled, highly proliferating cancer cells. The branching process is employed to consider the process of Type *S* proliferation (40).

Initially, *N* Type 0 cells occupy the tissue. There is a rare chance of a mutation every time a cell divides, and a daughter cell may change into a Type 1 cell with a mutation rate, *μ*_1_. Mutation rate, *μ*, refers to the sum total of the genomic or epigenetic factors affecting change from one cell type to another (41). When a cell dies, a cell to be divided in a tissue is selected depending on the cell fitness, *r*. The fitness of Type 0, Type 1, Type *K*, and Type *S*-1 are denoted by *r*_0_, *r*_1_, *r_K_* and *r_S_*_-1_, respectively. Cell fitness, *r* refers to the transcriptional and metabolic potential of a cell type to “out-compete” other cell types (42). A cell could divide to give rise to the same cell type or mutate to another cell type. When a Type 1 cell divides with a mutation, a daughter cell may change into a Type *K* cell with mutation rate *μ_K_* (if more mutations are needed) or a Type *S*-1 cell with a mutation rate, *μ_S_*_-1_ (if additional mutation steps are not needed). Intermediate cell type, Type *K*, becomes Type *S*-1 after sequential accumulation of mutations. Finally, a Type *S*-1 cell is capable of mutating to become a malignant cell – Type *S* cell based on the mutation rate from Type *S*-1 to Type *S* cell, *μ_S_*. Once a Type *S* cell appears, the cells proliferate indefinitely based on the growth rate of Type *S* cells, *r_S_*, disrupting tissue dynamics and homeostasis. Type *S* cancer cells are “super-competitors” with outstanding metabolic prowess and assumed to increase exponentially with a net growth rate of *r_S_* - *d_S_* > 0, where *d_S_* is a death rate.

We propose that the most important premalignant cells are the Type 1 cell that have acquired the first driver mutation and the Type *S*-1 cell that needs just one more driver mutation to become a cancer cell. These cells look phenotypically normal and are not regarded as important clinically but their genetic features are indispensable in cancer formation. As a result, the cell fitness of these 2 cell types must be taken into account in all computational analysis. For scenarios where additional mutations and more cell types are needed, we approximate intermediate mutational steps between Type 1 and Type *S*-1 by adjusting the values of *μ_S-1_* so that a low mutation rate, *μ_S-1_*, represents additional mutational steps. So, our computational analysis will be executed to account for the most important mutational events that affect cell fitness and all the mutation rates that can affect the number of steps required for carcinogenesis. In other words, we skip the state of Type *K* cell and a mutation rate stands in for the number steps.

The net growth of Type 0, Type 1, and Type *S*-1 cells is zero (equal frequency of cell division and death), while that of Type *S* cells is positive. Type 0, Type 1, and Type *S*-1 cells consist of a healthy tissue based on the Moran process, so *r_0_*, *r_1_* and *r_S-1_* are parameters to determine status of dividing cell and which daughter cell are obtainable at the time of a cell division. Alternatively, *r_S_* is the growth rate, which determines the average number of increases in Type *S* cells during a unit time. When the number of Type *S* cells reaches 10^9^ at the first time, all the Type *S* cells are discarded to represent surgical resection, whereas the number of Type 0, Type 1 and Type *S*-1 cells in a tissue is preserved so that the time until the emergence of the recurrent tumor is influenced by the frequency of residual Type 0, Type 1 and especially, Type *S*-1 cells. Since the conversion from the number of cells to the tumor volume is frequently done using the following relationship as 10^9^ cells in a 1 cm^3^ tumor, the time of surgery in this model is conducted when the size of the tumor becomes 1 cm^3^. We describe it as “time of cancer detection”. After the first treatment, the simulation continues until the next Type *S* cell appears from the tissue and the number reaches 10^9^ again, representing the recurrence of the tumor after surgery.

### Simulation framework

To integrate the Moran process and branching process, we adopted stochastic simulations based on Gillespie’s algorithm (43) as follows: We firstly considered three events: (i) cell turnover in a healthy tissue as per Moran process, (ii) birth of a Type *S* cell as per Branching process, and (iii) death of a Type *S* cell. The rates of each event at time *t* is given by (i) *dN* (ii) *r_S_X_S_* (*t*), and (iii) *d_S_X_S_* (*t*), respectively. Here *r_S_*, *d_S_*, and *X_S_* (*t*) are a proliferation rate, a death rate, and the number of Type *S* cells at time *t*, respectively. Then an average time until one of the three events happens, ∆T , is given by


$$∆T=\;\frac{1}{dN+r_{S}X_{S}\left(t\right)+d_{S}X_{S}\left(t\right)}.\;\;\;\;\;\;\;\;\;\;\;\;\;\;\;\;\left(1\right)$$

Let us first consider the case where a cell turnover happens. The probability that a cell turnover happens in ∆T is given by dN∙∆T . In our model, a cell turnover in a healthy tissue is governed by a cell death. When one of *N* cells is randomly selected as a cell to die, and another cell is chosen to divide within the same time step to complete cell turnover. In a healthy tissue, there are three types of cells, corresponding to the number of acquired mutations, Type 0, Type 1, and Type *S*-1.The number of each cell type is denoted by *X*_0_, *X*_1_ and *X_S_*_-1_, respectively. In brief, there are several possibilities of tissue composition transitions in the tissue dynamics and we consider the six events that affect the cell type composition of a tissue: (i) a type 0 cell increases by one while a type 1 cell decreases by one (ii) a type 0 cell increases by one while a type *S*-1 cell decreases by one (iii) a type 1 cell increases by one while a type 0 cell decrease by one (iv) a type 1 cell increases by one while a type *S*-1 cell decreases by one (v) a type *S*-1 cell increases by one while a type 0 cell decreases by one; or (vi) a type *S*-1 cell increases by one while a type 1 cell decreases by one. In such a condition, a Type 0 cell can increase by one if either a Type 1 or Type *S*-1 cell dies and a Type 0 cell divides without a mutation. Then the probability for these events leading to an increase in Type 0 cells are given by (i) $\frac{X_{1}}{N}∙\frac{r_{0}X_{0}\left(1-\mu_{1}\right)}{F}$ , and (ii) $\frac{X_{S-1}}{N}∙\frac{r_{0}X_{0}\left(1-\mu_{1}\right)}{F}$ . Here, $F=r_{0}X_{0}+r_{1}X_{1}+\;r_{S-1}X_{S-1}$ is a scaling factor for selecting a dividing cell. The probability of a Type 1 or Type *S*-1 cell death is given by $\frac{X_{1}}{N}$ and $\frac{X_{S-1}}{N}$ , respectively. Taken together, the transition probability that the number of Type 0 cell increases by one and that of Type 1 decreases by one is given by


$$\mathrm{Pr}[X_{0}\;\to \;X_{0}+1\;\text{and}\;X_{1}\;\to \;X_{1}-1\;]=\frac{X_{1}}{N}∙\frac{r_{0}X_{0}\left(1-\mu_{1}\right)}{F},\;\;\;\;\;\;\;\;\;\;\left(2\right)$$

and the probability that the number of Type 0 cell increases by one and that of Type *S*-1 decreases by one is given by


$$\mathrm{Pr}[X_{0}\;\to \;X_{0}+1\;\text{and}\;X_{S-1}\;\to \;X_{S-1}-1\;]=\frac{X_{S-1}}{N}∙\frac{r_{0}X_{0}\left(1-\mu_{1}\right)}{F}.\;\;\;\left(3\right)$$

A Type 1 cell can increase by one if either a Type 0 or Type *S*-1 cell dies, and either a Type 1 cell divides without mutation or a Type 0 cell divides with mutation to become a Type 1 cell. Then the probabilities for these events leading to an increase in Type 1 cells are given by (iii) $\frac{X_{0}}{N}∙\frac{r_{1}X_{1}\left(1-\mu_{S-1}\right)+r_{0}X_{0}\mu_{1}}{F}$ , and (iv) $\frac{X_{S-1}}{N}∙\frac{r_{1}X_{1}\left(1-\mu_{S-1}\right)+r_{0}X_{0}\mu_{1}}{F}$ . Taken together, the transition probability that the number of Type 1 cell increases by one and that of Type 0 decreases by one is given by


$$\mathrm{Pr}[X_{1}\;\to \;X_{1}+1\;\text{and}\;X_{0}\;\to \;X_{0}-1\;]=\frac{X_{0}}{N}∙\frac{r_{1}X_{1}\left(1-\mu_{S-1}\right)+r_{0}X_{0}\mu_{1}}{F},\;\;\;\left(4\right)$$

and the probability that the number of Type 1 cell increases by one and that of Type *S*-1 decreases by one is given by


$$\mathrm{Pr}[X_{1}\;\to \;X_{1}+1\;\text{and}\;X_{S-1}\;\to \;X_{S-1}-1\;]=\frac{X_{S-1}}{N}∙\frac{r_{1}X_{1}\left(1-\mu_{S-1}\right)+r_{0}X_{0}\mu_{1}}{F}.\;\;\left(5\right)$$

Similarly, a Type *S*-1 cell can increase by one if either a Type 0 or Type 1 cell dies, and either a Type *S*-1 cell divides without mutation or a Type 1 cell divides with mutation. The probabilities for the events leading to an increase in Type *S*-1 cells are given by (v) $\frac{X_{0}}{N}∙\frac{r_{S-1}X_{S-1}\left(1-\mu_{S}\right)+r_{1}X_{1}\mu_{S-1}}{F}$ , and (vi) $\frac{X_{1}}{N}∙\frac{r_{S-1}X_{S-1}\left(1-\mu_{S}\right)+r_{1}X_{1}\mu_{S-1}}{F}$ . Taken together, the transition probability that the number of Type *S*-1 cell increases by one and that of Type 0 decreases by one is given by


$$\mathrm{Pr}[X_{S-1}\;\to \;X_{S-1}+1\;\text{and}\;X_{0}\;\to \;X_{0}-1\;]=\frac{X_{0}}{N}∙\frac{r_{S-1}X_{S-1}\left(1-\mu_{S}\right)+r_{1}X_{1}\mu_{S-1}}{F},\;\;\left(6\right)$$

and the probability that the number of Type *S*-1 cell increases by one and that of Type 1 decreases by one is given by


$$\mathrm{Pr}[X_{S-1}\;\to \;X_{S-1}+1\;\text{and}\;X_{1}\;\to \;X_{1}-1\;]=\frac{X_{1}}{N}∙\frac{r_{S-1}X_{S-1}\left(1-\mu_{S}\right)+r_{1}X_{1}\mu_{S-1}}{F}.\;\left(7\right)$$

In addition, a Type *S* cell can increase by one if a Type *S*-1 cell divides with mutation. The probability is given by $\frac{r_{S-1}X_{S-1}\mu_{S}}{F}$ . Since a Type *S* cell is not a component of a tissue, once a Type *S* appears by mutation, another round of selection for a dividing cell is performed according to the transition probabilities described above. This is because malignant Type *S* cell disrupts 2D lattice structure and the Moran process is no longer applicable to it.

Next, let us consider the case where Type *S* cell divides or dies. The probabilities of Type *S* cell division or death is given by $r_{S}X_{S}\left(t\right)∙∆T$ and $d_{S}X_{S}\left(t\right)∙∆T$ , respectively.

In summary, the time of one step in our simulation is calculated using Eq. (1) and in one time step, one of the following three processes occurs: (i) a cell turnover in a tissue, (ii) the birth of a Type *S* cell, or (iii) the death of a Type *S* cell. Initially, all the cells are Type 0. Once the number of Type *S* cells reaches 10^9^, computational surgical resection sets the number of Type *S* cells to be 0, keeping the cell type composition in a tissue remained and computational carcinogenic process restarts again. After that, the time until the number of Type *S* cells reaches 10^9^ is measured as recurrence time.

### Spatial structure

Two-dimensional lattice structure (I×J) is introduced to a tissue dynamics in our computational model. The transition probabilities are basically the same with or without spatial structure. The difference is the choice of a dividing cell. If a cell at position 〈i,j〉 dies, 4 adjacent cells – 〈i,j−1〉 , 〈i,j+1〉 , 〈i−1,j〉 and 〈i+1,j〉 can divide to replace it. The transition probabilities are calculated according to the cell type at those positions. We assume wall boundary condition to represent an asymmetric tissue structure.

### Deterministic approximation of type *S* cell growth

As for the calculation of the Type *S* growth, we assume that when the number of cells is small, the stochastic effect should be considered. When the number of Type *S* cells exceed twice as large as the size of the normal tissue, 2*N*, growth can be regarded as a deterministic process. Then, the time duration from when the number of Type *S* cells is 2*N* to 10^9^, ∆*t*_s_, is given by


$$∆t_{s}=\;\frac{1}{\left(r_{S}-d_{S}\right)}\ln(\frac{10^{9}}{2N})\;\left(8\right)$$

### Clinical data

The data used in our analysis were from TCGA Pan-Cancer Clinical Data Resource (34, 35) and are available in the cBio Cancer Genomics Portal (44, 45). We adopt the clinical data of locoregional recurrence from 8,957 patients with 27 different non-sarcoma, non-hematological cancer types. From these datasets, the inclusion criterium for our study was “disease-free” survival – patients with no detectable malignant disease after surgery or total remission. We excluded data of “progression-free” survival in order to eliminate patients who survived with detectable disease possibly as a result of treatment-resistant clones; and also excluded data containing metastatic progression. We also included data from other independent publications for extra validation. Sarcomas and hematological cancers were excluded due to their non-conformity to a 2-dimensional lattice structure.

### Survival time analysis

Survival time analysis of clinical data is calculated using the Kaplan–Meier method from disease-free intervals mentioned in Clinical Data section. In this study, disease-free interval is defined as the survival time without cancer recurrence of each patient, which corresponds to the time to recurrence of each simulation trial.

### Statistical analysis

The whole process of our model is conducted on C++. Simulation codes have been deposited in a GitHub repository (https://github.com/sharaf501/Heano-Lab-Codes). The survival time analysis and other statistical analysis is conducted on Prism (version 9.4.1). Mantel-Cox (log-rank) test is used to compare difference between survival curves. A *p* value less than 0.05 is considered to be statistically significant.

## Results

### Cancer initiation patterns

**Figure 2:**
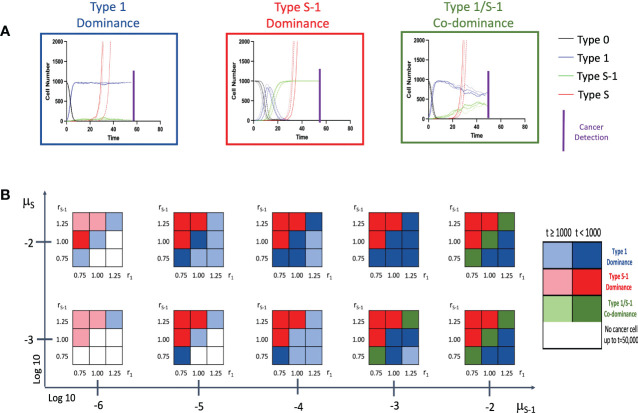
Patterns of tissue composition at cancer initiation **(A)** Simulation studies without spatial structure show three patterns of cancer initiation. For each pattern, black, blue, green and red curves indicate Type 0, Type 1, Type *S*-1 and Type *S* cells, respectively. Each parameter set was simulated in triplicate (Joined, dashed, and long-dashed lines). **(B)** Panel showing several patterns of tissue composition and time to detection using combination of various parameter sets. Cell type “Dominance” indicates >90% of a particular cell type at cancer detection. “Co-dominance” refers to 2 cell populations with >40% or 3 cell populations with >30% at cancer detection. Parameter values used are: *N* = 1,000; *d* = *d_s_* = 1.0; *r*_0_ = 1.0; *r*_1_ = 0.75, 1.00 and 1.25; *r_S_*_-1 =_ 0.75, 1.00 and 1.25; *r_S_* = 1.5; *μ*_1_ = 0.001 and 0.01; *μ_S_*_-1_ = 0.000001, 0.00001, 0.0001, 0.001 and 0.01; *μ_S_* = 0.001 and 0.01. Note: Mutation rate, *μ*_1_, does not impact the results and is not shown.

Firstly, we conducted stochastic triplicate simulations for the cancer initiation up to the time of cancer detection. We were curious to know what effect the presence or absence of the spatial structure would have on the model. We traced the time course of 4 cell populations – Type 0, Type 1, Type *S*-1 and Type *S* cells using a combination of various parameter sets. Lower mutation rate from Type 1 to Type *S*-1, *μ_S_*_-1_, was additionally examined to account for additional premalignant cell types between Type 1 and Type *S*-1. In the model without spatial structure, we observed 3 patterns of cancer initiation based on frequency of non-malignant cell population at cancer detection (**Figure 2A**). Interestingly, all the patterns show a progressive decline of Type 0 cells until the entire tissue is dominated by Type 1, Type *S*-1 or both Type 1 and Type *S*-1 cells. By combining various parameter sets in our simulation, we extrapolated the varying distribution of the cancer initiation patterns (**Figure 2B** and **Supplementary Figure 1**). Lower fitness of Type 1 cells, *r*_1_ generally favored Type *S*-1 cells dominance when fitness of Type *S*-1, *r_S_*_-1_, is high. Higher *r*_1_ values favored Type 1 dominance while equal fitness of Type 1 and Type *S*-1 cells yielded Type 1 dominance or Type 1/*S*-1 co-dominance. Mutation rates generally affected time to cancer detection and appearance of dominance. We also extended the mutation rates from Type 1 to Type *S*-1 cell type to denote other additional mutation steps and found a consistent increase in cancer detection times but patterns generally remained the same. In some cases where low fitness of both Type 1 and Type *S*-1 were coupled with lower mutation rates, Type *S* malignant cells failed to appear at extended times and simulations were terminated. Most curiously, mutation rate from Type 0 to Type 1, *μ*_1_, did not affect the pattern of cancer initiation or time to cancer detection (**Supplementary Figure 1**).

### Parameter dependence of recurrence time

**Figure 3:**
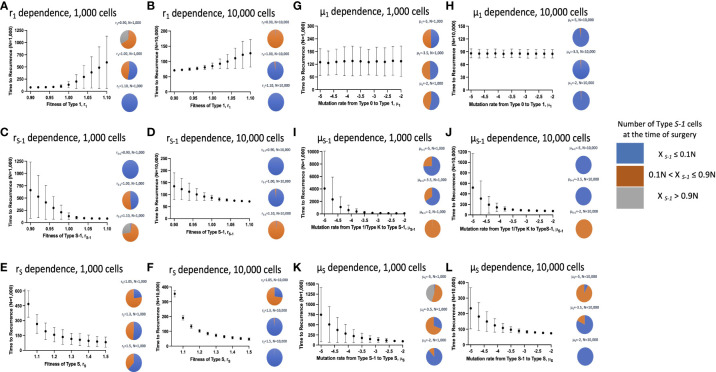
Parameter dependence on recurrence time. Simulation studies without spatial structure are shown. Mean values obtained from 100 to 1,000 simulations are shown by dots, and standard deviations are indicated by bars. Pie charts in the panels indicate the proportion of Type *S*-1 cells in a normal tissue at the time of first treatment. Blue, orange and grey represent small (*X_S_*_-1_ ≤ 0.1*N*), intermediate (0.1*N*< *X_S_*_-1_ ≤ 0.9*N*), and large (*X_S_*_-1_ > 0.9*N*) proportion of Type *S*-1 cells, respectively. Standard parameter values used in **(A–L)** are *d* = *d_s_* = 1.0, *r*_0_ = 1.0, *r*_1_ = 1.0, *r_S_*_-1_ = 1.2, *r_S_* = 1.0, *μ*_1_ = 0.001, *μ_S_*_-1_ = 0.001, *μ_S_* = 0.001; and *N* = 1,000 in **(A, C, E, G, I, K)**; and *N* = 10,000 in **(B, D, F, H, J, L)**.

Next, we examined the time to recurrence after surgical resection and the proportion of Type *S*-1 cells at the time of surgery in varying parameter sets. We reasoned that since Type *S*-1 cells needs only one more step for malignant transformation; therefore, its proportion was thought to be critical for cancer recurrence. To do this, we ran 1,000 simulations for each parameter set and calculated the mean recurrence time (**Figures 3A–K**). We also ran similar simulations at higher cell number in a tissue, *N*, between 100 to 1,000 times to assess the effect of tissue size on the parameter dependency (**Figures 3B–L**). We found that higher fitness of Type 1 cells, *r*_1_ increased the mean recurrence time (**Figures 3A, B**), while mutation rate from Type 0 to Type 1, *μ*_1_, had no effect on mean recurrence time (**Figures 3G, H**). Other parameters however, showed a negative correlation to the mean recurrence time – higher parameter values resulted in shorter mean recurrence time. Higher tissue cell number yielded an overall shortening of mean recurrence time but parameter dependency remained the same. We also observed a reduction in the proportion of Type *S*-1 cells at the time of surgery when *r*_1_, *r_S_* and *μ_S_* increases (**Figures 3A–L**), while *r_S_*_-1_ and *μ_S_* increases in the proportion of Type *S*-1 cells (**Figures 3C–J**). Mutation rate from Type 0 to Type 1, *μ*_1_, had little effect on the proportion of Type *S*-1 cells (**Figures 3G, H**).

### Effect of spatial structure on cancer initiation patterns

**Figure 4:**
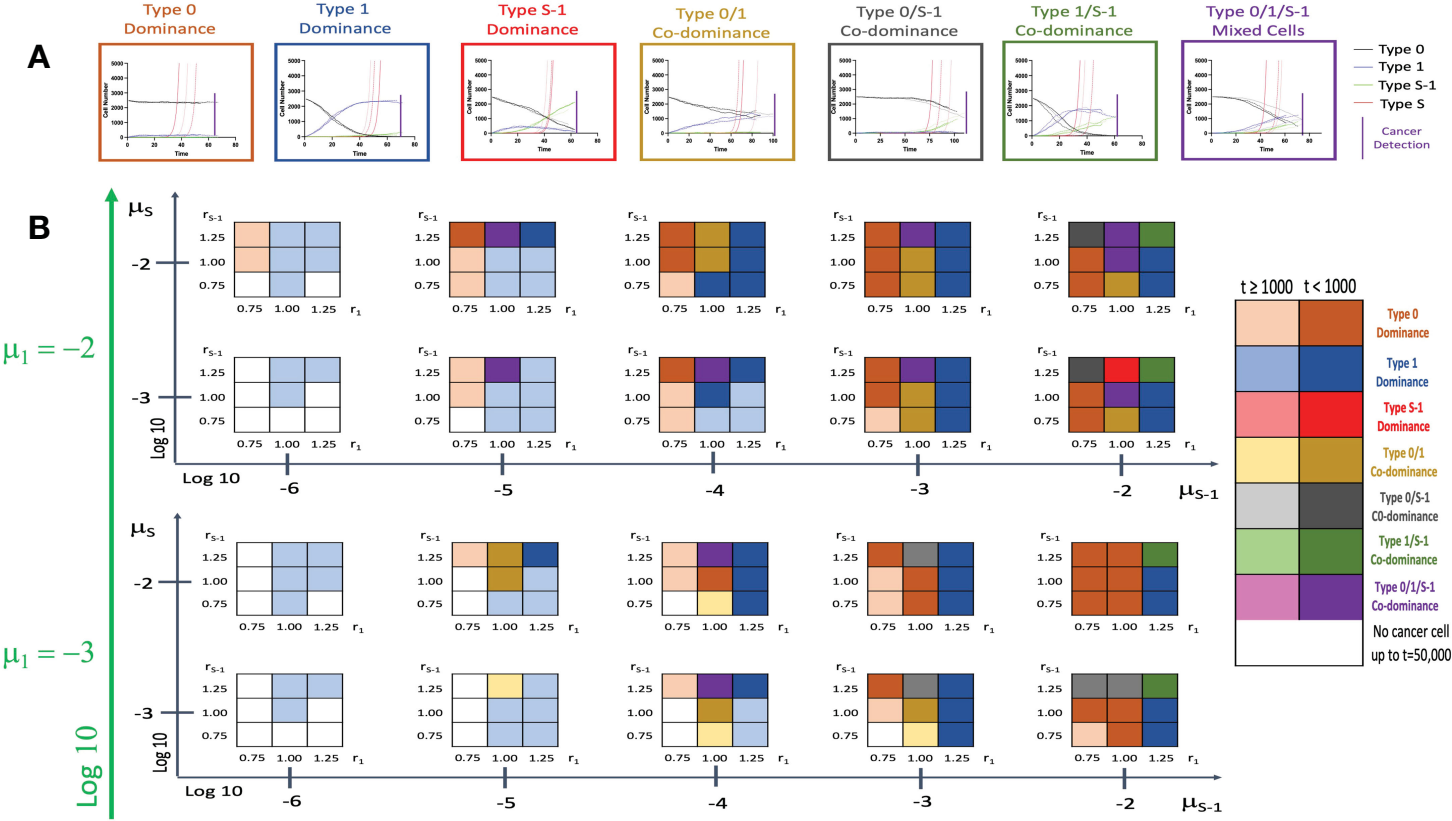
Patterns of tissue composition at cancer initiation with spatial structure. **(A)** Simulation studies with spatial structure show 7 patterns of cancer initiation. For each pattern, black, blue, green and red curves indicate Type 0, Type 1, Type *S*-1 and Type *S* cells, respectively. Each parameter set was simulated in triplicate (Joined, dashed, and long-dashed lines). **(B)** Panel showing patterns of tissue composition and time to detection using combination of various parameter sets. The definitions of “Dominance” and “Co-dominance” are the same as those explained in **Figure 2**. Parameter values used are: *N* = 2,500; *d* = *d_s_* =1.0; *r*_0_ = 1.0; *r*_1_ = 0.75, 1.00 and 1.25; *r_S_*_-1 =_ 0.75, 1.00 and 1.25; *r_S_* = 1.5; *μ*_1_ = 0.001 and 0.01; *μ_S_*_-1_ = 0.000001, 0.00001, 0.0001, 0.001 and 0.01; *μ_S_* = 0.001 and 0.01.

We then incorporated the spatial structure framework into the model to investigate the effect of tissue positional influence in cancer initiation patterns and time of cancer detection. After triplicate simulations using various parameters, we identified seven distinct patterns of cancer initiation (**Figure 4A**) based on the composition of non-malignant cell population at cancer detection. **Figure 4B** and **Supplementary Figure 2** showed the distribution of the patterns in a wide parameter range. Low *r*_1_ values showed Type 0 dominance at low *r_S_*_-1_ levels with failure to detect cancer cells at very low *μ_S_*_-1_ levels. With higher *r*_1_ values, Type 1 cell types begin to dominate. When combined with high *μ_S_*_-1_, we saw Type 1/*S*-1 co-dominance (green region in **Figure 2B**). When *r*_1_ and *r_S_*_-1_ are equal to fitness of Type 0 (*r*_0_), we saw Type 0/1 co-dominance or Type 0/1/*S*-1 co-dominance depending on *μ_S_*_-1_ values. We noticed a peculiar pattern of Type 0/*S*-1 co-dominance (black region in **Figure 2B**) when *r_S_*_-1_, *μ_S_*_-1_, and *μ_S_* were high with relatively lower *r*_1_ value. Type *S*-1 dominance (red region in **Figure 2B**) was regarded as the most undesirable scenario due to the abundance of Type *S*-1 cells, indicating shorter recurrence time. We saw this pattern when *r_S_*_-1_, *μ*_1_ and *μ*_S-1_ were high, *r*_1_ was equal to 1.0 and *μ*_S_ was relatively small. Some parameter sets with low fitness failed to yield Type *S* cells at extended time points during the simulations. Here, the incorporation of the spatial structure to our simulation framework had remarkable alterations to the cancer initiation patterns and cancer detection time.

### Effect of spatial structure on recurrence time

**Figure 5:**
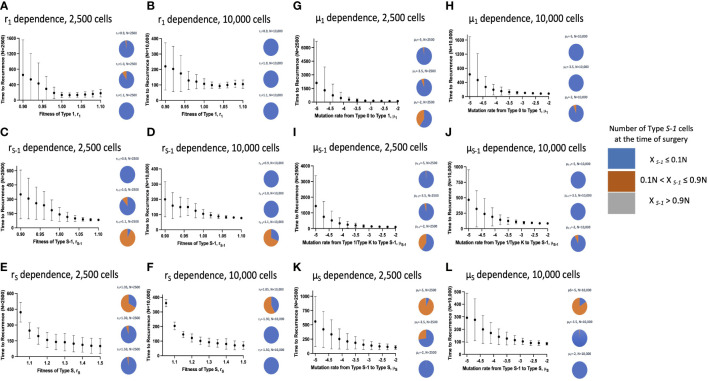
Parameter dependence on recurrence time with spatial structure. Simulation results with spatial structure are shown. Mean values obtained from 100 to 1,000 simulations are shown by dots, and standard deviations are indicated by bars. Pie charts in the panels indicate the proportion of Type *S*-1 cells in a normal tissue at the time of first treatment. Blue, orange and grey represent small (*X_S_*_-1_ ≤ 0.1*N*), intermediate (0.1*N*< *X_S_*_-1_ ≤ 0.9*N*), and large (*X_S_*_-1_ > 0.9*N*) proportion of Type *S*-1 cells, respectively. Standard parameter values used in **(A–L)** are *d* = *d_s_* =1.0, *r*_0_ = 1.0, *r*_1_ = 1.0, *r_S_*_-1_ = 1.2, *r_S_* = 1.0, *μ*_1_ = 0.001, *μ_S_*_-1_ = 0.001, *μ_S_* = 0.001; and *N* = 2,500 in **(A, C, E, G, I, K)**; and *N* = 10,000 in **(B, D, F, H, J, L)**.

Subsequently, we examined the mean recurrence time after surgical resection and the proportion of Type *S*-1 lesions at the time of surgery in a vast parameter range with the influence of the spatial structure setting (**Figure 5**). Similarly, we ran 100 to 500 simulations to obtain mean recurrence time (**Figures 5A–K**) and to check the effect of larger cell numbers (**Figures 5B–L**). Generally, we saw that the integration of the spatial structure to our simulation framework had noteworthy changes to the parameter dependency to recurrence time. When the size of the normal tissue was small, the effect of fitness advantage on the proportion of Type *S*-1 cells in a tissue became larger. Our simulation results showed that an increase in the cell fitness shortened the mean time to recurrence (**Figures 5C–F**). However, an increase in *r*_1_ was found to reduce the recurrence time but begin to increase slightly at much higher levels regardless of the tissue size. We also found a consistent reduction in the mean recurrence time as mutation rates *μ*_1_, *μ_S_*_-1_ and *μ_S_* increased (**Figures 5G–L**). We also observed a reduction in the proportion of Type *S*-1 cells at the time of surgery with a spatial structure. Especially, when either *r_S_* or *μ_S_* was small, and any of *r_S-_*_1_, *μ*_1_, or *μ_S-_*_1_was large, the proportion of Type *S*-1 increased (**Figure 5**).

### Fitting of recurrence time to clinical data

**Figure 6:**
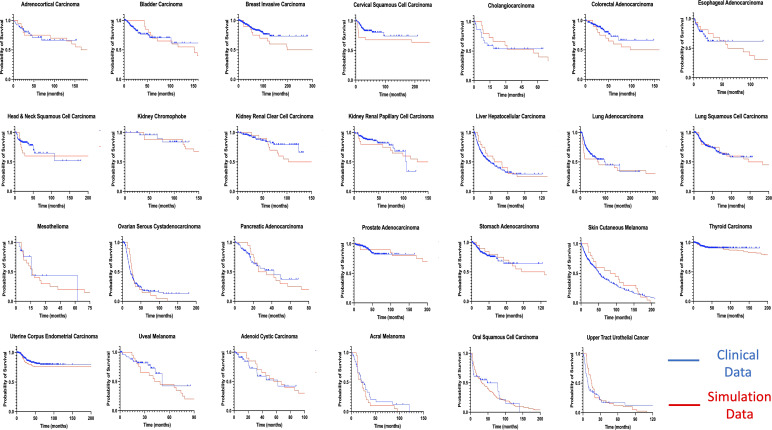
Fitting of model-derived *in silico* data to published clinical data for 27 cancer types. Thousands of stochastic runs were used to obtain parameter sets that best fit survival curves of 27 non-sarcoma, non-hematologic cancer types. Blue curves indicate clinical data while red curves indicate simulation data survival curves. *p* values between curves are found in **Table 1**. ACC, Adrenocortical Carcinoma; BLCA, Bladder Urothelial Carcinoma; BRCA, Breast Invasive Carcinoma; CESC - Cervical Squamous Cell Carcinoma; CHOL – Cholangiocarcinoma; COAD, Colorectal Adenocarcinoma; ESCA, Esophageal Adenocarcinoma; HNSC, Head & Neck Squamous Cell Carcinoma; KICH, Kidney Chromophobe; KIRC, Kidney Renal Clear Cell Carcinoma; KIRP, Kidney Renal Papillary Cell Carcinoma; LIHC, Liver Hepatocellular Carcinoma; LUAD, Lung Adenocarcinoma; LUSC, Lung Squamous Cell Carcinoma; MESO – Mesothelioma; OV, Ovarian Serous Cystadenocarcinoma; PAAD, Pancreatic Adenocarcinoma; PRAD, Prostate Adenocarcinoma; STAD, Stomach Adenocarcinoma; THCA, Thyroid Carcinoma; UCEC, Uterine Corpus Endometrial Carcinoma; UVM, Uveal Melanoma; ACYC, Adenoid Cystic Carcinoma; MEL, Acral Melanoma; UTUC, Upper Tract Urothelial Cancer; OSCC, Oral Squamous Cell Carcinoma; SKCM, Skin Cutaneous Melanoma.

By using our computational model with spatial structure, multiple runs of stochastic simulations were performed with multiple parameter sets and *in silico* Kaplan–Meier curves were made. The data points about the time when 0% to 100% of patients experienced recurrence with an interval of 4% (time when 0%, 4%, 8%, …, 100% of patients experienced recurrence) were employed to compare between the *in silico* and published clinical data (44, 45) of 27 cancer types. In this analysis, we adopted random sampling for parameters to obtain *in silico* recurrence data and determined the best parameter set for each cancer type that minimized the mean of squared logarithmic residuals (log-MSR) between outputs *in silico* and in public. The accepted parameter set (**Table 1**) was used to extrapolate recurrence time which were then fitted to clinical data and disease-free survival curves were depicted (**Figure 6**).

**Table 1:** Tumor-specific carcinogenic profiles and p values of survival curves (**µ** values are in log_10_ while SQ are log-SSR values).

Code	Cancer Type (Data Source)	SQ	r_1_	r_S-1_	r_S_	d_S_	µ_1_	µ_S-1_	µ_S_	µ_I_	p value
ACC	Adrenocortical Carcinoma (TCGA, PanCancer Atlas)	0.616	1.003	0.958	5.578	3.436	-3.212	-3.979	-3.006	-10.197	0.9120
BLCA	Bladder Urothelial Carcinoma (TCGA, PanCancer Atlas)	0.922	1.097	0.981	5.614	3.939	-2.876	-3.616	-3.223	-9.715	0.8857
BRCA	Breast Invasive Carcinoma (TCGA, PanCancer Atlas)	0.983	0.945	0.948	2.967	1.887	-3.770	-2.228	-2.661	-8.658	0.1707
CESC	Cervical Squamous Cell Carcinoma (TCGA, PanCancer Atlas)	0.897	0.929	0.951	6.684	3.506	-3.842	-3.233	-2.098	-9.172	0.1213
CHOL	Cholangiocarcinoma (TCGA, PanCancer Atlas)	0.847	0.988	1.011	7.170	3.765	-3.504	-2.759	-2.349	-8.612	0.7716
COAD	Colorectal Adenocarcinoma (TCGA, PanCancer Atlas)	0.943	1.086	1.027	5.912	3.859	-3.176	-3.052	-4.347	-10.575	0.2783
ESCA	Esophageal Adenocarcinoma (TCGA, PanCancer Atlas)	0.936	1.048	0.919	6.767	3.655	-4.114	-3.870	-2.411	-10.394	0.8862
HNSC	Head & Neck Squamous Cell Carcinoma (TCGA, PanCancer Atlas)	0.802	0.980	0.988	5.148	3.586	-2.735	-4.886	-2.656	-10.277	0.5908
KICH	Kidney Chromophobe (TCGA, PanCancer Atlas)	0.401	1.070	1.024	2.308	1.558	-3.971	-2.542	-4.658	-11.170	0.9360
KIRC	Kidney Renal Clear Cell Carcinoma (TCGA, PanCancer Atlas)	0.786	1.081	1.064	3.655	2.713	-4.569	-3.445	-4.252	-12.265	0.1519
KIRP	Kidney Renal Papillary Cell Carcinoma (TCGA, PanCancer Atlas)	0.559	0.965	0.958	5.470	3.004	-3.182	-2.748	-3.374	-9.304	0.9880
LIHC	Liver Hepatocellular Carcinoma (TCGA, PanCancer Atlas)	0.846	0.977	1.009	7.675	3.899	-4.620	-3.690	-4.252	-12.562	0.6744
LUAD	Lung Adenocarcinoma (TCGA, PanCancer Atlas)	0.600	0.922	0.977	6.383	3.327	-3.068	-3.471	-2.821	-9.360	0.4484
LUSC	Lung Squamous Cell Carcinoma (TCGA, PanCancer Atlas)	0.524	0.997	0.952	5.220	3.568	-4.174	-2.276	-3.258	-9.708	0.9767
MESO	Mesothelioma (TCGA, PanCancer Atlas)	0.572	0.918	1.030	7.134	3.581	-3.287	-4.222	-3.561	-11.070	0.8411
OV	Ovarian Serous Cystadenocarcinoma (TCGA, PanCancer Atlas)	0.772	1.052	0.939	4.145	2.655	-3.483	-2.415	-3.502	-9.400	0.9710
PAAD	Pancreatic Adenocarcinoma (TCGA, PanCancer Atlas)	0.736	0.988	0.996	3.145	1.654	-3.218	-2.875	-2.416	-8.509	0.8931
PRAD	Prostate Adenocarcinoma (TCGA, PanCancer Atlas)	0.886	0.915	0.918	4.826	3.584	-2.286	-3.987	-2.189	-8.462	0.7336
STAD	Stomach Adenocarcinoma (TCGA, PanCancer Atlas)	0.400	0.936	0.949	5.738	3.177	-3.377	-2.268	-3.389	-9.034	0.9442
SKCM	Skin Cutaneous Melanoma (TCGA, Firehose Legacy)	0.805	1.016	0.980	2.848	1.818	-4.481	-3.607	-2.512	-10.601	0.5770
THCA	Thyroid Carcinoma (TCGA, PanCancer Atlas)	0.764	1.020	0.980	6.541	3.525	-2.824	-4.268	-4.131	-11.222	0.7340
UCEC	Uterine Corpus Endometrial Carcinoma (TCGA, PanCancer Atlas)	0.892	0.948	0.984	3.965	2.083	-4.276	-3.824	-2.908	-11.007	0.1153
UVM	Uveal Melanoma (TCGA, Firehose Legacy)	0.564	1.022	0.947	6.612	3.535	-3.397	-2.166	-4.000	-9.563	0.2663
ACYC	Adenoid Cystic Carcinoma (MSK, Nat Genet 2013)	0.521	1.082	1.065	4.523	2.492	-2.754	-3.342	-4.046	-10.142	0.6316
MEL	Acral Melanoma (TGEN, Genome Res 2017)	0.524	1.031	1.008	5.199	3.104	-2.572	-3.866	-2.487	-8.925	0.3385
OSCC	Oral Squamous Cell Carcinoma (MD Anderson, Canc. Disc 2013)	1.033	0.901	0.990	8.090	3.700	-2.721	-2.602	-3.640	-8.964	0.9997
UTUC	Upper Tract Urothelial Cancer (MSK, Eur Urol 2015)	0.628	0.938	1.009	7.470	3.800	-2.130	-3.672	-2.815	-8.617	0.5480

**Figure 7:**
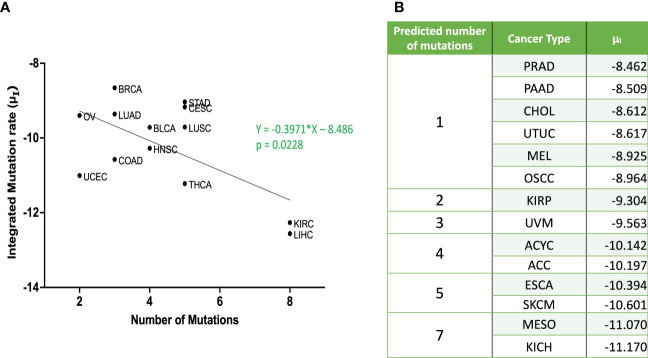
Relationship between integrated mutation rate and number of mutation hits required for cancer initiation **(A)** Published data for number of mutational hits required for carcinogenesis (37) in some cancer types was plotted against corresponding integrated mutation rate $\left(\mu_{1}∙\mu_{S-1}∙\mu_{S}\right)$ . The linear regression was performed, and the regression line and the *p* value are shown. **(B)** Predicted number of mutations for unpublished cancer types as per equation from **(A)** ACC, Adrenocortical Carcinoma; BLCA, Bladder Urothelial Carcinoma; BRCA, Breast Invasive Carcinoma; CESC, Cervical Squamous Cell Carcinoma; CHOL – Cholangiocarcinoma; COAD, Colorectal Adenocarcinoma; ESCA, Esophageal Adenocarcinoma; HNSC, Head & Neck Squamous Cell Carcinoma; KICH, Kidney Chromophobe; KIRC, Kidney Renal Clear Cell Carcinoma; KIRP, Kidney Renal Papillary Cell Carcinoma; LIHC, Liver Hepatocellular Carcinoma; LUAD, Lung Adenocarcinoma; LUSC, Lung Squamous Cell Carcinoma; MESO – Mesothelioma; OV, Ovarian Serous Cystadenocarcinoma; PAAD, Pancreatic Adenocarcinoma; PRAD, Prostate Adenocarcinoma; STAD, Stomach Adenocarcinoma; THCA, Thyroid Carcinoma; UCEC, Uterine Corpus Endometrial Carcinoma; UVM, Uveal Melanoma; ACYC, Adenoid Cystic Carcinoma; MEL, Acral Melanoma; UTUC, Upper Tract Urothelial Cancer; OSCC, Oral Squamous Cell Carcinoma; SKCM, Skin Cutaneous Melanoma.

Mantel-Cox test was used to compare between the curves of simulated and clinical data revealing minimal statistical nonconformity. According to the estimated parameters (**Table 1**), we firstly deduced a tissue-specific turnover per month from *d_S_*. Kidney chromophobe had the fewest cellular turnover cycles per month while bladder urothelial carcinoma and colorectal adenocarcinoma had the highest turnover cycles. Moreover, colorectal adenocarcinoma, kidney chromophobe, renal clear cell carcinoma, thyroid carcinoma, adenoid cystic carcinoma and acral melanoma showed higher fitness of all their premalignant cells than normal cells. Of note, the proliferation rate of the Type *S* malignant cells, *r_S_*, was estimated to be high in cholangiocarcinoma, liver hepatocellular carcinoma, mesothelioma and upper tract urothelial cancer while being relatively low in breast invasive carcinoma, kidney chromophobe and skin cutaneous melanoma. Kidney chromophobe had the lowest mutation rate from the final premalignant cell stage to malignant cells while cervical squamous cell carcinoma and prostate adenocarcinoma had the highest mutation rate. **Figure 7** showed the negative correlation between mutational steps required for carcinogenesis (37) and overall mutation rates (*μ_I_*) obtained from our studies by multiplying the mutation rates for all steps.

## Discussion

In this study, we constructed computational models with and without spatial structure that described cell population dynamics in both normal and cancer tissues. Using our models, we clearly observed different patterns of cancer initiation and the residual premalignant cells present at the time of cancer detection or surgical intervention. Integrating the spatial structure setting to the model revealed additional patterns of cancer initiation as against just three in the model without spatial structure. Especially, the preservation of intact normal cells was observed in the model with spatial structure (**Figures 2**–**4**). According to the comprehensive analysis of parameter dependence, we found that field cancerization at the detection time depended on a combination of fitness and mutation rates.

We also revealed the relationship between the proportion of premalignant cells and recurrence time (**Figures 3**–**5**). The model without spatial structure overemphasized the power of Type 1 fitness and its ability to limit Type *S*-1 and Type *S* appearance which led to longer mean recurrence times as *r*_1_ increases (**Figures 3A, B**). The same effect was seen in the mutation rate from Type 0 to Type 1 which rendered *μ*_1_ impotent in affecting mean recurrence time (**Figures 3G, H**). All other fitness and mutation parameters led to shorter recurrence time as their effects became larger. Generally, with the spatial structure setting, we found that recurrence time became shorter when mutation rates or fitness of cancer cells were large, while the time became longer when the fitness of premalignant cells or growth rate of cancer cells were low (**Figure 5**). An exception would be the mean recurrence time with *r*_1_ (**Figures 5A, B**) which was seen to shorten as *r*_1_ became larger but to get longer as *r*_1_ became much larger. This could be due to a tissue competition between Type 1 and Type *S*-1 cells which subsequently delayed the emergence of Type *S* cells and hence, a more favorable recurrence time.

Moreover, we successfully estimated the characteristic parameter sets of the computational model that best reproduced the clinical data of disease-free survival in each cancer type. All the non-sarcoma cancer types were successfully fitted with no statistical deviation (**Table 1**). Even though some datasets like ESCA contains 2 different cancer types – esophageal adenocarcinoma and esophageal squamous cell carcinoma, we obtained *p* values that indicates no statistical difference. At the same time, we obtained valuable information about cellular turnover per month (*d_S_*), relative fitness of premalignant cells (*r*_1_, *r_S_*_-1_), a growth rate of cancer cells (*r_S_*) and mutation rates from one cell type to another (*μ*_1_, *μ_S_*_-1_, *μ_S_*) for each carcinogenesis. We have specified the growth rate for each cancer using the *r*_S_ values from our clinical fitting. Interestingly, we observed relatively high growth rates of malignant cells (Type *S *cells) in some common cancer types like lung and colorectal cancers, whereas a relatively lower growth rate was estimated in breast invasive carcinoma which was also a common cancer type but was relatively asymptomatic in agreement with several studies (46, 47). From the high *μ_S_*_-1_ values, we elucidated that uveal melanoma, breast invasive carcinoma, stomach adenocarcinoma and lung squamous cell carcinoma had the shortest time to reach late premalignant cell stage from the earliest premalignant cell stage possibly indicating fewer mutational steps. On the other hand, thyroid carcinoma and head and neck carcinoma had small *μ_S_*_-1_ values, indicating the multiple steps in the carcinogenesis. Our data was in alignment with data that estimated the number of hits required for carcinogenesis (37), where liver, kidney and thyroid cancers had the lowest overall mutational rates indicating more mutational requirements while uterine, ovarian and lung cancers had higher overall mutational rates indicating fewer mutational requirements (**Figure 7**).

Additionally, our findings successfully revealed average cellular turnover rates per month by inferring our model with published clinical data whose measurements were in months. Kidney chromophobe and pancreatic cancer showed low turnover rates per month (about 1.5 times), possibly indicating low incidence rate. On the other hand, bladder urothelial carcinoma, liver hepatocellular carcinoma, colorectal adenocarcinoma and upper tract urothelial carcinoma had the highest turnover rates (almost 4 times per month) which perhaps explained why they were the most common cancers in men and women (48). This also corresponded with data that suggested that number of cell division was a significant risk factor for cancer (49).

We propose from our findings that certain cell populations, specifically Type S-1 could be targeted to address the threat of locoregional recurrence. With currently available tools and advancements in personalized medicine, it is possible to prevent recurrence by targeting a particular cell type or lesion. An example in case in the outstanding success achieved using PD-1 Blockade in mismatch repair–deficient, locally advanced rectal cancer which recorded a 100% success (50). CRISPR-based mutation can also aid in cell competition studies to identify cell fitness levels among the known and unknown driver mutations to further provide actionable data for more studies.

In this study, we estimated cell fitness as a single numerical value with 1.0 indicating normal cells and other cells with ranges from normal cells. In reality, this is an “oversimplification” as cell fitness is a complex and dynamic concept which can be related to both genetic (51) and non-genetic (52) alterations. Unfortunately, studies on cell fitness with regards to known or even unknown cancer-related mutations are lacking. Also, order of mutations in premalignant cells and a comprehensive study of cell-based or animal model mutational requirements for certain cancers are unavailable. These limit the tools with which we can perform additional validation of our model. Mutation rates were chosen to include processes involved with DNA repair, epigenetics, infection and role of external agents. Each of these could independently affect the model but we chose to combine them. In the current analysis, hematologic or liquid cancers were not included partly because of their dynamic nature and lack of 2D lattice arrangement but mainly the difficulty in assessing exact cell numbers. Even though certain tumor markers for certain malignancies may be used to quantify cell number, the threshold for detection and overall utility is not fully assured. The model without spatial structure might be applicable in this scenario as well as for sarcomas. Moreover, we did not stratify or independently differentiate demographic information such as age, sex or race for each cancer type. Possible extension of the analysis may be to perform age or other parameter dependent analysis. Furthermore, we did not specify the order of mutations for malignant transformation in our model, which albeit gave us a good fit with clinical data. The order of mutations is quite important as revealed from data accrued from colorectal cancer progression (53). Considering multiple mutational orders could be beneficial especially those leading to histologically ‘abnormal’ benign lesions. Barrett’s Esophagus (BE) is a notable example where whole genome sequencing found similar mutational events between esophageal adenocarcinoma and non-dysplastic BE (54) thereby suggesting different mutational order (55). Reports that prior diagnosis of BE affords a better prognosis (56) with only about 5% of BE patients developing esophageal adenocarcinoma (57) further strengthens the different order of mutation concept.

One challenge for cancer management is late diagnosis. Our model computes a cancer detection stage of 1cm^3^ – 10^9^ cells. To evaluate the effect of late diagnosis, we changed the cancer detection time to 10^10^ and assess parameter dependence on recurrence time. We observed a reduction in time to recurrence indicating that late diagnosis might contribute to shorter recurrence time (**Supplementary Figure 3**). Another challenge to the usage of this model is the variability of proportion of locoregional recurrence out of total recurrence rate among various cancer types. It is common knowledge that recurrence can occur at a distant area from the original issue – metastasis; our model however, does not take this into account. As a result, the utility of this model is high for certain cancer types but unfortunately, subdued for other cancer types. Consequently, malignancies where locoregional recurrence accounts for a high proportion of total recurrence such as thyroid cancer with 94% (58), oral squamous cell carcinoma with 90% (59), cholangiocarcinoma with 85% (60), prostate cancer with 81% (61), liver cancer with 78% (62), mesothelioma with 74% (63), head & neck squamous cell carcinoma with 69% (64), and ovarian cancer with 68% (65) could reap great benefit from this model. On the other hand, cancers where distant metastasis accounts for a major proportion of total recurrence such as kidney cancers with 73% (66), skin cutaneous melanoma with 71% (67) and bladder urothelial cancer with 66% (68) might feel the need to complement our model with additional tools to increase its precision. Interestingly, we can get some insight from recurrence pattern of breast cancer. In patients undergoing conservative breast surgery only, locoregional recurrence accounts for 62% of all cancer recurrence (69). However, in a study with data for different surgical intervention types, locoregional recurrence rates were 42.9% and 19% of total recurrence in breast conservative surgery and total mastectomy respectively (70). This could perhaps be due to the elimination of the cancerized field by total mastectomy which conservative surgery is unable to achieve.

In conclusion, this model reveals parameter combinations that fit clinical data and contributes to the ever-growing knowledge about cancer initiation and recurrence. The model shows elucidate cancers which have premalignant cells with high fitness are likely to have a short recurrence time. This approach can be a valuable tool in the management of cancer especially in the field of personalized molecular medicine to target patients who are at highest risk of recurrence.

## Data availability statement

The datasets presented in this study can be found in online repositories. The names of the repository/repositories and accession number(s) can be found in the article/**Supplementary Material**.

## Author contributions

SDA, MT, and HH conceived the idea of the study. SDA, MT, and HH developed simulation codes. SDA conducted computational simulations. SDA and HH contributed to the interpretation of the results. HH supervised the conduct of this study. All authors reviewed the manuscript draft and revised it. All authors approved the final version of the manuscript to be published. 
