# Supplementary material for: Computational modeling of locoregional recurrence with spatial structure identifies tissue-specific carcinogenic profiles
Source: Front Oncol. 2023 Apr 6;13:1116210. doi: 10.3389/fonc.2023.1116210 (PMC10117647; doi:10.3389/fonc.2023.1116210)
Supplement: Supplementary file 1 [file DataSheet_1.docx]

**Supplementary Figure 1**

**Extended Cancer Initiation Pattern without spatial Structure for Figure 2 with (a) low r_1_/r_S-1_ (b) low r_1_/normal r_S-1_ (c) low r_1_/ high r_S-1_ (d) normal r_1_/ low r_S-1_ (e) normal r_1_/r_S-1_ (f) normal r_1_/ high r_S-1_ (g) high r_1_/low r_S-1_ (h) high r_1_/normal r_S-1_ and (i) high r_1_/r_S-1_ at different mutation rates.**

**Supplementary Figure 2**

**Extended Cancer Initiation Pattern with spatial Structure for Figure 4 with (a) low r_1_/r_S-1_ (b) low r_1_/normal r_S-1_ (c) low r_1_/ high r_S-1_ (d) normal r_1_/ low r_S-1_ (e) normal r_1_/r_S-1_ (f) normal r_1_/ high r_S-1_ (g) high r_1_/low r_S-1_ (h) high r_1_/normal r_S-1_ and (i) high r_1_/r_S-1_ at different mutation rates.**

**Supplementary Figure 3**

**Effect of late diagnosis on cancer recurrence time. Using the model with spatial structure, parameter dependence with spatial structure was done with late diagnosis where cancer detection was done at 10^10^ cells (right panel) rather than 10^9^ cells (left panel)**

**Supplementary Figure 1**

**(a)**

**(b)**

**(c)**

**(d)**

**(e)**

**(f)**

**(g)**

**(h)**

**(i)**

**Supplementary Figure 2**

**(a)**

**(b)**

**(c)**

**(d)**

**(e)**

**(f)**

**(g)**

**(h)**

**(i)**

**Supplementary Figure 3**
